# Unraveling Metabolite Provisioning to Offspring Through Parental Fluids: A Case Study of the Brazilian Guitarfish, *Pseudobatos horkelii*

**DOI:** 10.3389/fphys.2022.911617

**Published:** 2022-06-20

**Authors:** Natascha Wosnick, Renata Daldin Leite, Eloísa Pinheiro Giareta, Danny Morick, Rachel Ann Hauser-Davis

**Affiliations:** ^1^ Programa de Pós-graduação em Zoologia, Universidade Federal do Paraná, Curitiba, Brazil; ^2^ Morris Kahn Marine Research Station, University of Haifa, Haifa, Israel; ^3^ Department of Marine Biology, Leon H. Charney School of Marine Sciences, University of Haifa, Haifa, Israel; ^4^ Hong Kong Branch of Southern Marine Science and Engineering, Guangdong Laboratory (Guangzhou), Hong Kong, Hong Kong SAR, China; ^5^ Laboratório de Avaliação e Promoção da Saúde Ambiental, Instituto Oswaldo Cruz, Fundação Oswaldo Cruz (Fiocruz), Rio de Janeiro, Brazil

**Keywords:** conservation physiology, elasmobranch, metabolism, reproduction, viviparity

## Abstract

Elasmobranchs have a very distinct metabolism, and many aspects related to the energetic dynamics of these animals remain poorly investigated. The reproductive period is particularly vulnerable for viviparous species, as part of the energy reserves of the parental biomass is reallocated for gamete production and embryo development. In this context, this study aimed to characterize parental metabolite provisioning to the offspring (both sperm and developing embryos) of the Brazilian Guitarfish, *Pseudobatos horkelii*, through glucose, β-hydroxybutyrate, triglycerides, and total cholesterol determinations in the uterine liquid (UL) and serum of pregnant females and in the seminal fluid (SF) and serum of males during the copulation period. No significant difference was observed for the analyzed markers between the UL and SF. Except for triglycerides, higher in female serum samples, all other energy markers were present at similar concentrations in the serum of both females and males. When comparing female UL and serum, significant differences were observed for triglycerides and total cholesterol. No differences were observed between SF and serum in males. The results indicate that all markers are being made available to offspring, possibly complementary to the yolk in the case of maternal liquid, and as an additional source for sperm mobilization required during egg fertilization in the case of the paternal fluid. Correlations between the markers in the parental matrices were also noted, compatible with the metabolic pathways activated during energy mobilization in vertebrates. Moreover, distinct marker predominance patterns were also noted for both UL and SF. Energy mobilization characterization directed to offspring through parental fluids aids in unraveling metabolic dynamics during the reproduction stage while also providing support for stress physiology studies to evaluate the indirect effects of parental allostatic overload in both sperm and developing embryos. Finally, energy mobilization assessments of parental fluids may also help elucidate how internal fertilization and viviparity evolved in this very distinct taxonomic group.

## Introduction

Elasmobranchs have a very unusual metabolism, differing from the pattern observed for most vertebrates (Ballantyne, 2015). Due to their urotelic osmoregulatory strategy, energy mobilization in this group is directed to maintain high concentrations of circulating urea, directly affecting the nitrogen metabolism and energy intake dynamics. Sharks and rays rely on a protein/lipid-rich diet, with little or no dependence on carbohydrates as an energy source (Speers-Roesch and Treberg, 2010). As they do not have adipose tissue, all energy reserves are directed to the hepatic tissue and, secondarily, to muscle tissue (Ballantyne, 1997). Despite presenting lower protein assimilation rates compared to teleost fish, evolutionary adaptations that facilitate amino acid intake are observed, including the expression and secretion of digestive enzymes in the most basal lineages (Ballantyne, 2015) and the ability to reduce stomach pH to values much lower than those observed in other vertebrates (Wood et al., 2007). As for lipid assimilation, elasmobranchs also present a set of physiological adaptations to ensure proper lipid intake, including certain emulsifying agents present in bile exclusive to this group (Ballantyne, 1997). As a result, ketone bodies comprise the main energy fuel for this taxonomic group, obtained both from lipid oxidation and the restricted dietary intake of carbohydrates, significantly reducing the role of glucose as a metabolic fuel.

Hepatic storage is an important adaptation to ensure energy mobilization when needed, allowing for rapid ketone body uptake as a source of oxidizable carbon to other tissues in specific situations (i.e., fight or flight responses). In female elasmobranchs, the liver also plays an imperative role in offspring nutrition, as vitellogenesis depends on hepatic reserve energy mobilization (Rossouw, 1987). Such is the importance of energy fuel storage in this tissue that some behaviors, such as migration, can only be performed when reserves are preserved (Del Raye et al., 2013), which may partly explain why females tend to move less and approach the coast when pregnant. In fact, the reproductive period is very costly for sharks and rays, as part of the dietary intake is directed to gamete production and offspring maintenance. In viviparous species, the cost of reproduction is so elevated that studies indicate higher parental mortality upon capture stress during the copulation (males) and embryonic development (females) phases, due to the inability of individuals to properly direct stored energy to deal with allostatic overload and recover both homeostatic and acid-basic balances (Wosnick et al., 2019; Prado et al., 2021).

Although parental input to embryo nutrition is well-established through the detailed description of vitellogenesis for various elasmobranchs (Ballantyne, 1997; Lucifora et al., 2002), several knowledge gaps still remain especially for other parental matrices, including uterine fluid in the case of viviparous species (i.e., lecithotrophic) and uterine milk in the case of matrotrophic viviparous species (i.e., histotrophy). In the case of males, knowledge is even more incipient, as detailed semen descriptions are often restricted to sperm morphology, mobility, and spermatozoa concentrations (Girard et al., 2000; Dzyuba et al., 2019; Morales-Gamba et al., 2019; Rowley et al., 2019). As spermatozoa rely on external signaling to activate motility, it is reasonable to infer that some energy source may be available in the seminal fluid to complement the energy expenditure necessary during sperm transfer and egg fertilization. The same can be inferred for developing embryos, as the uterine liquid may serve as a complementary energy source to the yolk sac, therefore, playing an additional role apart from embryonic gas exchanges and osmotic balance. In fact, Lombardi et al. (1993) first suggested that maternal nutrient transfer during gestation in viviparous elasmobranchs involves passage from uterine tissues to embryonic absorptive cell types, and that metabolite incorporation in embryos would involve uptake by tissues bathed by periembryonic/uterine fluids. Some Rhinobatidae species have been postulated as utilizing uterine liquid enriched with mucus, fat or protein as a complementary form of nutrition (Dulvy and Reynolds, 1997), although this has not been studied in detail to date for all species belonging to this group, including Brazilian guitarfishes. In fact, Vooren et al. (2005) indicate that this species displays a viviparous lecithotrophic mode of reproduction, and, furthermore, may be classified as exhibiting incipient matrotrophy, with low levels of maternal input, indicative of a mid-evolutionary sequence ranging from low to high yolk only nutrition, according to Dulvy and Reynolds (1997).

Some physiological markers have been employed to access energy mobilization in vertebrates. Glucose, for example, is the most abundant monosaccharide, being stored as glycogen. It is a ubiquitous source of energy that fuels both aerobic and anaerobic cellular respiration in both invertebrates and vertebrates (Gerich, 2000). Nevertheless, its role as a metabolic fuel in sharks and rays is not yet fully understood. Another physiological marker comprises the ketone body β-hydroxybutyrate, an important metabolic substrate utilized in energy production during nutrient deprivation, produced primarily from acetyl CoA by liver mitochondria and then exported to peripheral tissues for oxidation, also modulating nutrient utilization and energy expenditure through signaling functions in several metabolic pathways or processes (Ballantyne 1997; Rojas-Morales et al., 2016). Increased body β-hydroxybutyrate levels in the blood have been reported as indicative of both higher muscular activity and mobilization of lipid reserves (Ballantyne, 1997; Speers-Roesch and Treberg, 2010). Cholesterol, on the other hand, is a precursor for the synthesis of steroid-based hormones, bile acids, and vitamin D, and plays a main role in maintaining cell membrane integrity and fluidity (Zampelas and Magriplis, 2019). It is also a useful lipid reserve use biomarker, alongside triacylglycerol, another physiological marker, the major form in which fat energy is stored, hydrolyzed in the gut by lipases to fatty acids and monoglycerides (Kaçar, 2019). Increases in the levels of both of these compounds in elasmobranchs have been reported as indicating lipid reserve mobilization associated with reproduction (García-Garrido et al., 1990; Pethybridge et al., 2014).

The present study aimed to characterize parental metabolite provisioning directed to offspring (both spermatozoa and developing embryos) employing the Brazilian Guitarfish, *Pseudobatos horkelii*, a lecithotrophic viviparous species (Vooren et al., 2005) as a study model through glucose, β-hydroxybutyrate, triglycerides, and total cholesterol determinations in the uterine liquid (UL), seminal fluid (SF) coupled to parental serum evaluations. To this end, six hypotheses were empirically tested. First, that significant concentrations of the aforementioned energetic markers will be detected in both UL and SF, indicating a fuel source for gamete mobilization during fertilization and an additional nutritional source for embryos that rely on yolk sac reserves during development. Second, all markers will be observed at similar concentrations in parental serum, indicating a similar energy source during the reproductive period for both sexes. Third, markers will also be observed in similar concentrations in both UL and SF, indicating similar importance for spermatozoa and embryos. Fourth, all markers will be detected in similar concentrations in both parental serum and reproductive matrices, indicating that parental serum is the main source of metabolite provisioning in the species. Fifth, correlations between markers will be observed considering metabolic dynamics previously described for vertebrates (e.g., the link between glucose and triglycerides during gluconeogenesis). Sixth, β-hydroxybutyrate will be the most relevant marker, while glucose will be of least significance, based on the energy metabolism pattern traditionally described for elasmobranchs.

## Methods

### Animal Collection

Pregnant female and sexually active male (i.e., caught during the copulation period) *P. horkelii* individuals incidentally caught by artisanal fishers in the states of Paraná and Rio de Janeiro (Southeastern and Southern Brazil, respectively) were donated to our research group. The animals were caught in bottom gillnets in fishing campaigns lasting about 12 h. Only recently deceased guitarfish were sampled (score 1 for all categories), using a freshness index, considering the following variables: overall gill color (1 for reddish and 0 for pinkish or whitish coloration), ocular retraction level (1 for non-retracted and bright and 0 for retracted and opaque), blood clotting (1 for unclotted and 0 for partially or fully clotted), and *rigor-mortis* (1 for complete absence and 0 for partial or complete presence). Prior to necropsies, the individuals were measured (total length–TL in cm) and sexed (males—*n* = 10; TL–83.9 ± 4.4 cm; females—n = 8; TL–105.7 ± 11.5 cm; embryos with total length ranging from 210 to 240 mm). Samplings were approved by the Brazilian Ministry of Environment (IBAMA/ICMBio-SISBIO #70981-3 and # 77310-3).

### Parental Fluid Sampling

In pregnant females, abdominal incisions were performed using a sterile scalpel and scissors and the uterine liquids were collected from the left uteri using a 26 G needle attached to a 1 ml disposable syringe. The samples were immediately transferred to sterile polypropylene microcentrifuge tubes (2 ml), stored in a polystyrene box containing ice, and taken to the laboratory. Upon arrival, the samples were frozen at −20°C until analysis. Sperm was collected from sexually active adult males through manual clasper pressure (1 ml) according to García-Salinas et al. (2021). The samples were then immediately transferred to sterile polypropylene microcentrifuge tubes (2 ml) and transported and stored using the same protocol described for UL.

### Serum Sampling

Following UL and SF sampling, blood samples (2 ml) were obtained by caudal venipuncture using a 22 G needle attached to a 3 ml disposable syringe, and immediately transferred to ultra-pure polypropylene microcentrifuge tubes (2 ml). The blood samples were immediately stored in a polystyrene box with ice and taken to the laboratory. Upon arrival, the blood samples were centrifuged for 7 min at room temperature (20°C) at 2,000 g. The serum samples were then separated and frozen at −20°C until analysis.

### Energetic Marker Assays

All samples were thawed and homogenized prior to the analyses. To evaluate metabolite provisioning to offspring and sperm through parental fluids, glucose, β-hydroxybutyrate, triglycerides, and total cholesterol were quantified in technical triplicates for each sample. Glucose (catalog n. 113; wave-length 505 nm), triglycerides (catalog n. 87; wave-length 505 nm), and total cholesterol (catalog n. 76; wave-length 500 nm) were assayed colorimetrically (Kasuaki IL-120 UV-Vis Spectrophotometer) using Labtest kits (Labtest, 2022). β-hydroxybutyrate was quantified using a FreeStyle Optium (FSO, Abbott) portable device, following validation for non-human vertebrates (Fiorentin et al., 2017; Ribeiro et al., 2021). All assays were carried out strictly following the manufacturer’s instructions and with previously sterilized material to avoid contamination.

### Statistical Analyses

Initially, pairwise tests were performed (Student’s t-test or Wilcoxon test) to compare the determined energetic markers between sexes (uterine liquid vs. seminal fluid; male serum vs. female serum). Pairwise tests were also performed comparing male seminal fluid vs. male serum, and female uterine fluid vs. female serum. In order to identify whether the analyzed markers are correlated, Pearson or Spearman correlations were performed separately for the seminal fluid, uterine liquid and serum of each sex. Finally, in order to verify potential correlations between the values of each parameter analyzed in the seminal fluid or uterine liquid with serum concentrations, Pearson or Spearman correlations were also performed individually for each parameter between the two measurements. All tests were performed following normality and homogeneity assumptions tested with Shapiro-Wilk test and F test, respectively. All analyses were performed using the R environment, at a significance level of *p* < 0.05 (R Development Core Team, 2021).

## Results

**FIGURE 1:**
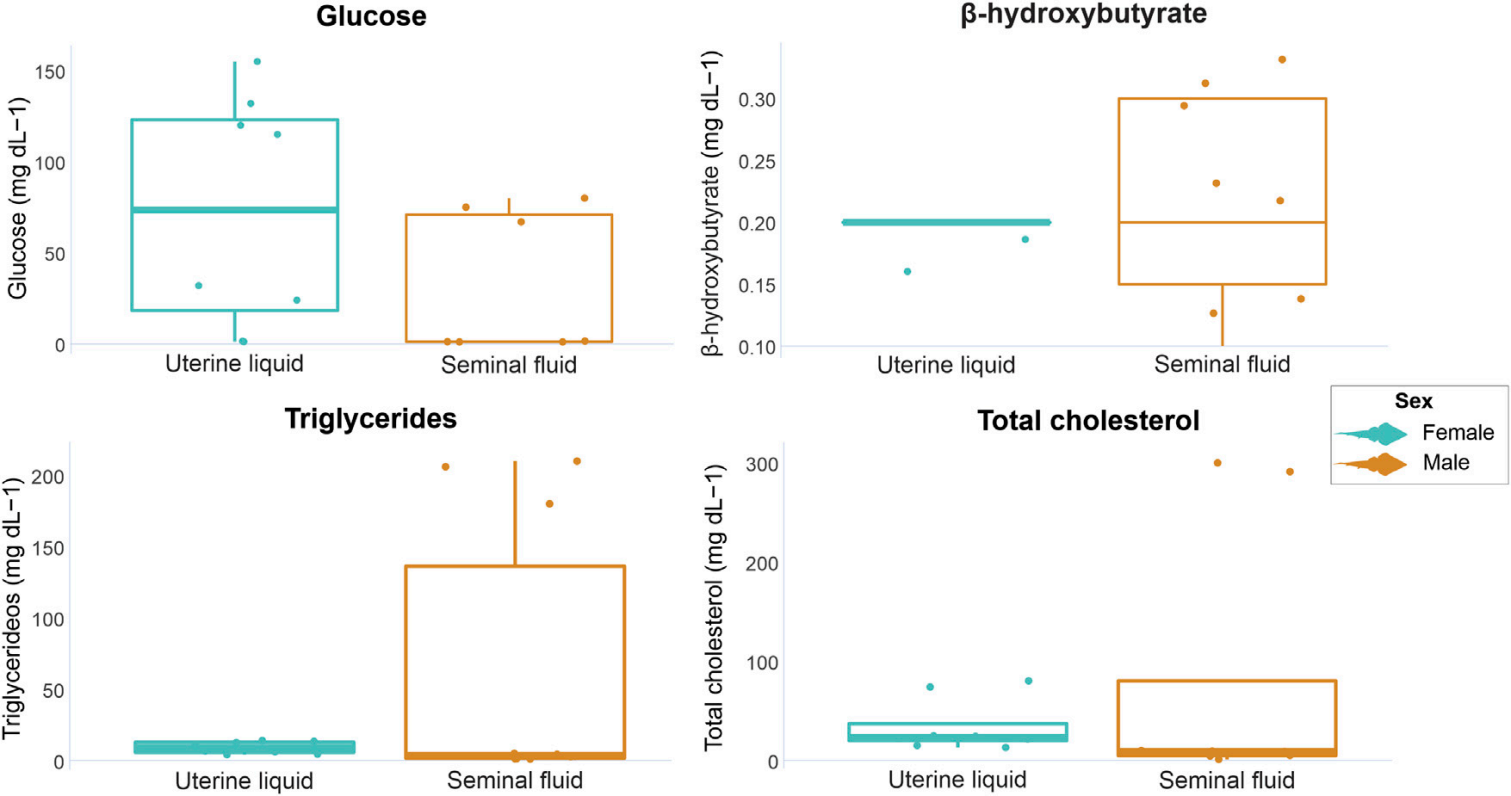
Graphical representation of *P. horkelii* energetic marker concentrations in the uterine liquid (UL) of pregnant females (light blue boxplots) and in the seminal fluid (SF) of sexually active males (during the copulation phase) (orange boxplots).

In the UL, glucose concentrations ranged from 1.2 to 155.0 mg dL-1, β-hydroxybutyrate from undetectable to 0.2 mg dL-1, triglycerides from 4.1 to 14 mg dL-1 and total cholesterol from 13.04 to 80.1 mg dL-1. In the SF, glucose concentrations ranged from undetectable to 80.0 mg dL-1, β-hydroxybutyrate from undetectable to 0.3 mg dL-1, triglycerides from 1.1 to 210.0 mg dL-1, and total cholesterol from undetectable to 300.0 mg dL-1. The minimum, maximum, mean values, and standard deviation of all analyzed markers are displayed in Table 1. No statistical differences concerning marker concentrations were observed between parental matrices (UL and SF) (glucose W = 42, *p* = 0.12; β-hydroxybutyrate t = −0.42008, df = 6, *p* = 0.68; triglycerides W = 53, *p* = 0.27; total cholesterol W = 48, *p* = 0.10). However, overall glucose concentrations were slightly higher in the UL compared to the SF, while β-hydroxybutyrate concentrations tended towards slightly higher in the SF when compared to the UL, albeit non-significantly (Figure 1). As for triglycerides, concentrations were similar in both parental matrices, but higher circulating levels were occasionally observed in the SF. Overall total cholesterol concentrations were also similar in the UF and the SL, with little variations within the analyzed individuals for each parental matrix (Figure 1).

**TABLE 1:** Minimum, maximum, and mean concentrations, and standard deviations of the analyzed seminal and uterine fluid *P. horkelii* markers. Concentrations are presented in mg dL^−1^.

		Minimum	Maximum	Mean ± SD
Uterine liquid (UL)	Glucose (mg dL-1)	1.2	155	72.58 ± 63.83
	β-hydroxybutyrate (mg dL-1)	0.2	0.2	0.2 ± 0
	Triglycerides (mg dL-1)	4.1	14	9.06 ± 4.13
	Total cholesterol (mg dL-1)	13.04	80.1	34.41 ± 26.65
Seminal fluid (SF)	Glucose (mg dL-1)	1.1	80	32.43 ± 39.07
	β-hydroxybutyrate (mg dL-1)	0.1	0.3	0.2 ± 0.08
	Triglycerides (mg dL-1)	1.1	210	61.43 ± 95
	Total cholesterol (mg dL-1)	1.1	300	78.73 ± 133.93

**FIGURE 2:**
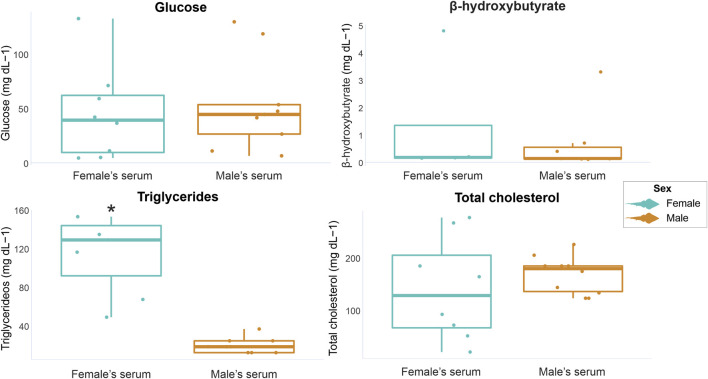
Graphical representation of *P. horkelii* energetic marker concentrations in the serum of pregnant females (light blue boxplots) and of sexually active males (during the copulation phase) (orange boxplots).

In female serum samples, glucose concentrations ranged from 4.5 to 132.5 mg dL-1, β-hydroxybutyrate from undetectable to 4.8 mg dL-1, triglycerides from undetectable to 153.06 mg dL-1 and total cholesterol from 20.51 to 276.92 mg dL-1. In male serum samples, glucose concentrations ranged from undetectable to 129.5 mg dL-1, β-hydroxybutyrate from undetectable to 3.3 mg dL-, triglycerides from undetectable to 36.73 mg dL-1 and total cholesterol from 123.07 to 225.64 mg dL-1. The minimum, maximum, mean values, and standard deviations of all analyzed markers are displayed in Table 2. When comparing marker concentrations in serum between sexes, a significant difference was observed for triglycerides (W = 63, *p* = 0.0001), while no significant differences were observed for the other parameters (glucose W = 32, *p* = 0.74; β-hydroxybutyrate W = 19, *p* = 0.41; total cholesterol t = −0.7501, df = 8.4997, *p* = 0.63) (Figure 2).

**TABLE 2:** Minimum, maximum, and mean concentrations, and standard deviations of the analyzed *P. horkelii* serum markers. Concentrations are presented as mg dL^-1^.

		Minimum	Maximum	Mean ± SD
Female's serum	Glucose (mg dL-1)	4.5	132.5	45.2 ± 43.09
	β-hydroxybutyrate (mg dL-1)	0.15	4.8	1.32 ± 2.31
	Triglycerides (mg dL-1)	48.97	153.06	114.57 ± 41.02
	Total cholesterol (mg dL-1)	20.51	276.92	141.02 ± 97.41
Male's serum	Glucose (mg dL-1)	6.5	129.5	53.22 ± 43.30
	β-hydroxybutyrate (mg dL-1)	0.11	3.3	0.7 ± 1.16
	Triglycerides (mg dL-1)	12.24	36.73	19.72 ± 7.96
	Total cholesterol (mg dL-1)	123.07	225.64	168.20 ± 35.59

**FIGURE 3:**
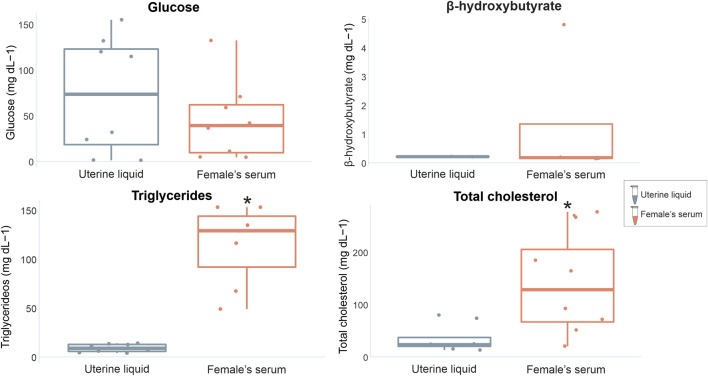
Graphical representation of *P. horkelii* energetic marker concentrations in the uterine liquid (UL) (grey boxplots) and serum of pregnant females (light pink boxplots).

**FIGURE 4:**
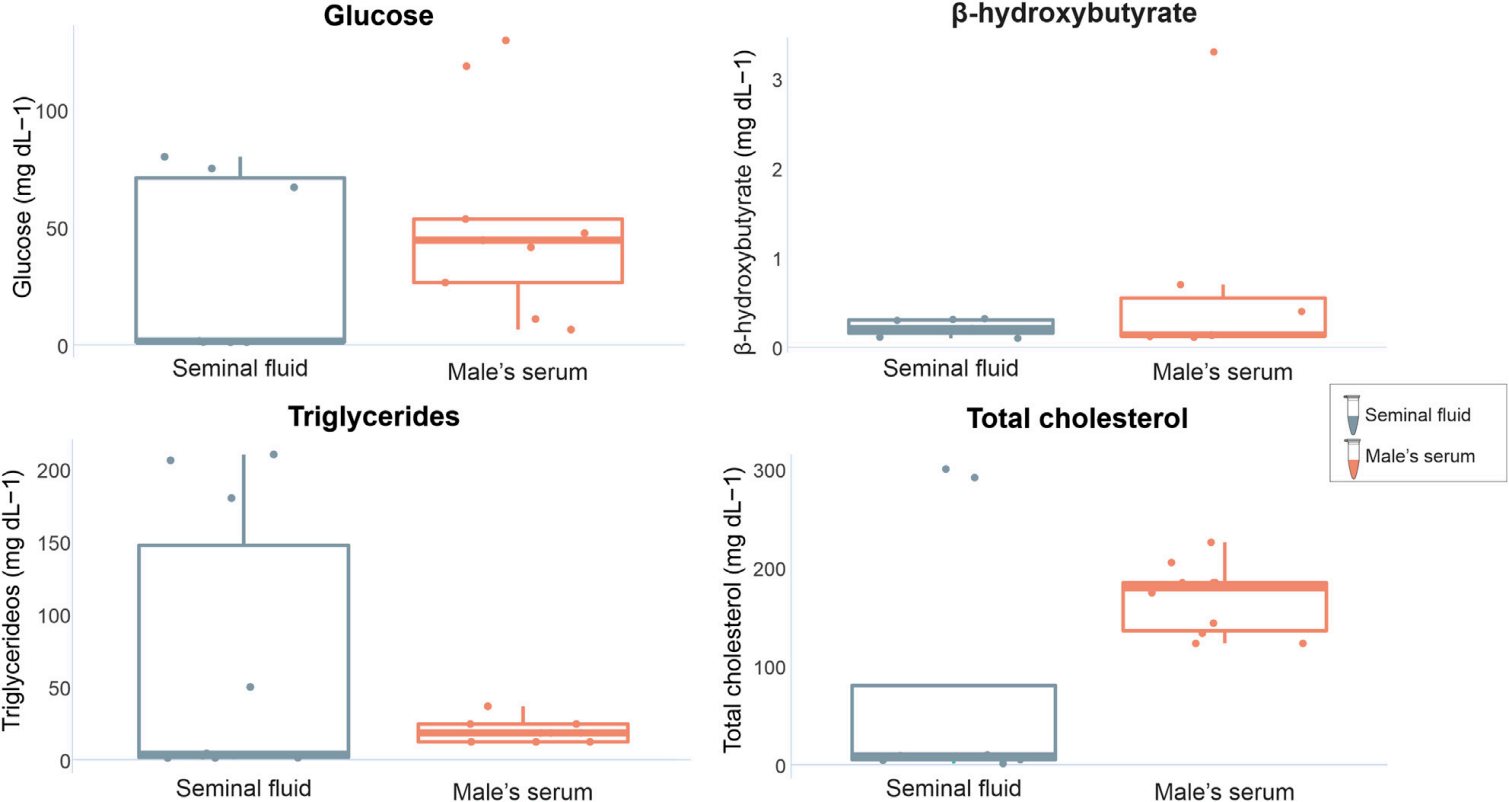
Graphical representation of *P. horkelii* energetic marker concentrations in the seminal fluid (SF) (grey boxplots) and serum of sexually active males (during the copulation phase) (light pink boxplots).

When comparing marker concentrations between uterine fluid and serum in females, significant differences were identified for triglycerides (W = 0, *p* = 0.0003) and total cholesterol (W = 10, *p* = 0.02), while glucose and β-hydroxybutyrate exhibited no significant differences (W = 35, *p* = 0.79 and W = 4 *p* = 1 respectively) (Figure 3). Comparisons between the analyzed markers in the seminal fluid and serum of males indicated non-significant differences (glucose t = −0.99309, df = 14, *p* = 0.33; β-hydroxybutyrate W = 21, *p* = 0.71; triglycerides W = 36, *p* = 0.49; total cholesterol W = 20, *p* = 0.08) (Figure 4).

**FIGURE 5:**
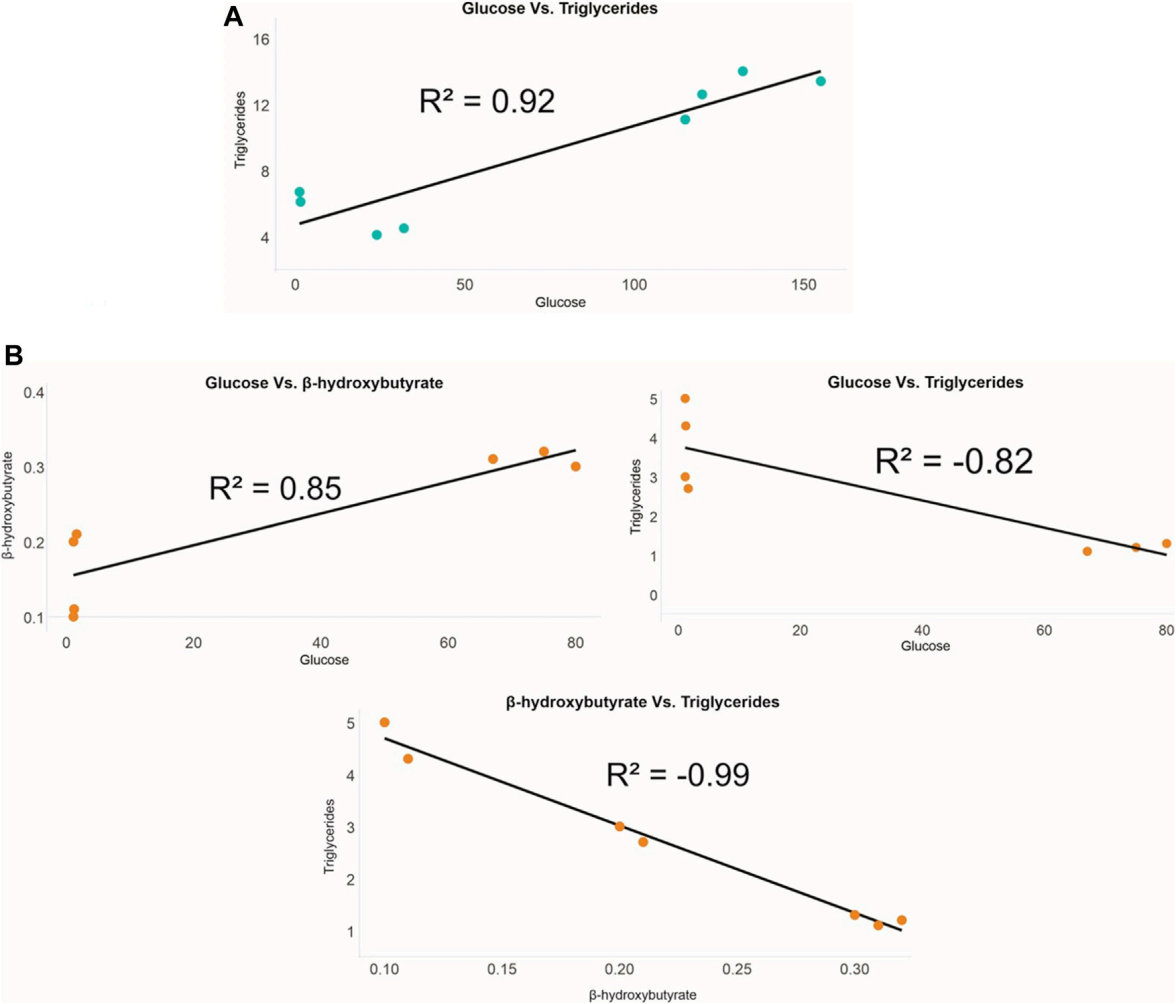
Correlations between *P. horkelii* energy markers in the **(A)** uterine liquid of pregnant females (blue) and in the **(B)** seminal fluid of sexually active males (orange).

In the UL of pregnant females, only a positive correlation between glucose and triglycerides (R^2^ = 0.92) was detected (Figure 5A). As for the SF of sexually active males, a positive correlation between glucose and β-hydroxybutyrate (R^2^ = 0.85) and negative correlations between glucose and triglycerides (R^2^ = −0.82) and between β-hydroxybutyrate and triglycerides (R^2^ = −0.99) were noted (Figure 5B). In serum, no significant correlations were detected between the analyzed markers for either females or males. Furthermore, no significant correlations were found when testing for correlations between the values of each analyzed marker between UL and serum (in females) and SF and serum (in males).

**FIGURE 6:**
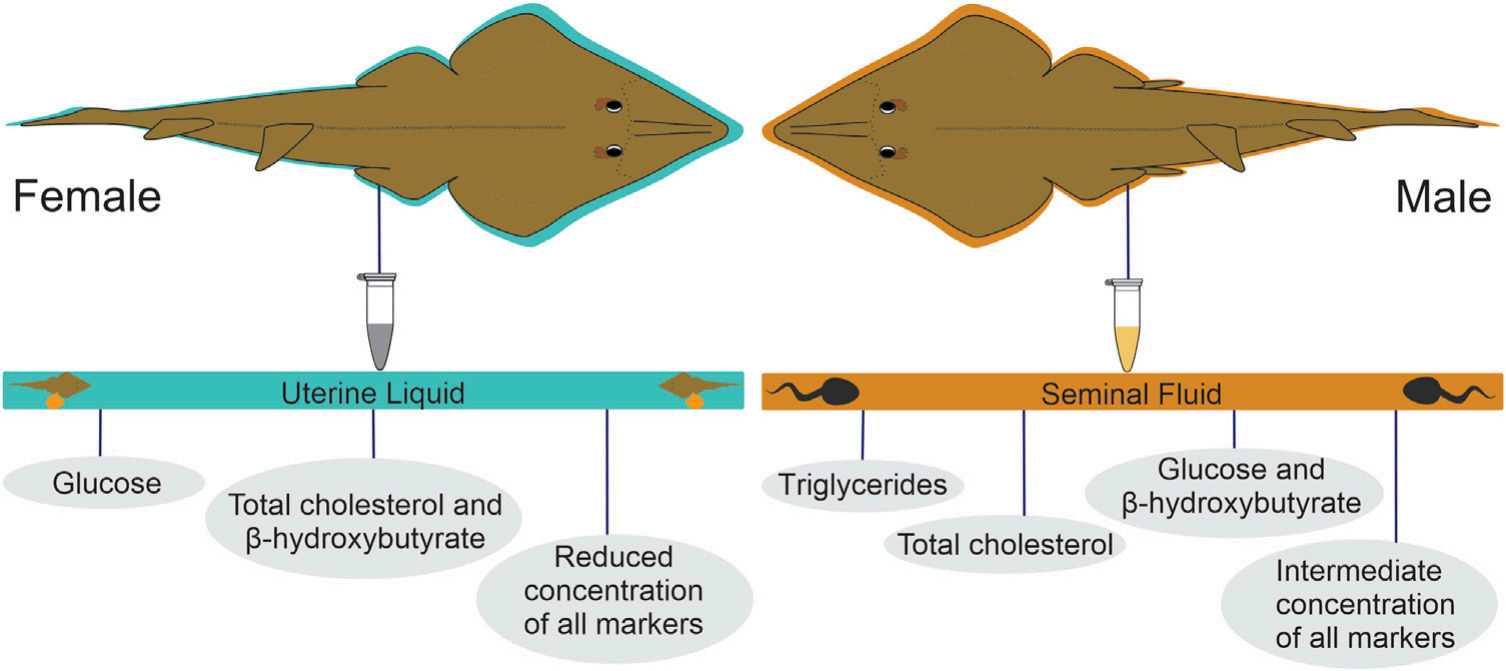
Metabolite provisioning patterns observed in the uterine liquid of pregnant females and in the seminal fluid of sexually active males of *P. horkelii*.

Finally, three distinct energy mobilization patterns were observed in the UL: 1) the predominance of glucose, 2) the predominance of β-hydroxybutyrate and total cholesterol, and 3) reduced concentrations for all analyzed substrates. As for the SF, four distinct patterns were identified: 1) the predominance of triglycerides, 2) the predominance of total cholesterol, 3) the predominance of glucose, and β-hydroxybutyrate, and 4) intermediate concentrations for all energy substrates (Figure 6).

## Discussion

We found support for our first hypothesis that significant concentrations of all markers would be detected in both the UL and SF. In lecithotrophic viviparous elasmobranchs, embryo development is internal and nutrition is performed through a yolk sac produced by the mother prior to oocyte fertilization, in a process called vitellogenesis. The embryos then remain inside egg capsules filled with intracapsular liquid (IL), an extension of maternal UL. While the main function of both IL and UL is to ensure gas exchange and osmo-ionic balance, there is increasing evidence that the UL also functions as a source of embryo nutrition, as in the case of great white sharks (*Carcharodon carcharias*) (Sato et al., 2016) prior to oophagy. In the case of the Grey Nurse Shark (*Carcharias taurus*) embryonic nutrition is even more complex, with at least six distinct nutrient assimilation phases, including the yolk sac, UL, and lastly, egg capsules containing other embryos (Gilmore, 1993). Although these aforementioned species are matrotrophic, lecithotrophic species may also present complimentary nutrition phases during pregnancy, such as energy markers (i.e., glucose, triglycerides, and total cholesterol), which have been previously detected in the UL of the Ornate Wobbegong (*Orectolobus ornatus*) (Ellis and Otway, 2011), the Spiny Dogfish (*Squalus acanthias*) and the Dusky Smoothound (*Mustelus canis*) (Lombardi et al., 1993). In the present study, glucose, β-hydroxybutyrate, triglycerides, and total cholesterol were detected in the UL of pregnant females. As the UL is formed from maternal serum (Price and Daiber, 1967; Kormanik, 1993), it is possible that the detected energy markers are being made available in the intrauterine environment as a way of providing additional nutrition to the embryos being nourished by the yolk. Although it is possible that the presence of these energy substrates in the UL is arbitrary (i.e., the ability to cross the uterine barrier, being present only as a consequence of circulating serum levels), the loss of these molecules through the UL would represent a significant debt to the female energy metabolism, as pregnancy already poses a challenge to adequate maternal nutrient assimilation. Further studies are necessary to better understand how energy markers in the UL behave at different pregnancy stages, i.e., if their role is in fact complementary to the yolk, or whether the UL becomes the main nutrition source after yolk assimilation in the final stages of embryo development. In addition, further studies on the provisioning of energy substrates from maternal serum to the UL are also necessary, as a possible evolutionary adaptation of the UL for embryo nutrition in lecithotrophic viviparous species.

Concerning the SF, information is scarce and normally focused on sperm morphology/motility rather than on environmental tonicity changes that take place during fertilization (Dzyuba et al., 2019; Rowley et al., 2019). The roles of lipids and fatty acids on sperm physiology have been previously described for the Ocellate River Stingray (*Potamotrygon motoro*), indicating that low concentrations on sperm membranes may be the main reason for their low tolerance to hypotonicity (Dzyuba et al., 2019). In teleost fish, the biochemical evaluation of seminal plasma is an important criterion for the assessment of milt quality (Billard et al., 1995), and semen characteristics vary from species to species (Chowdhury and Joy, 2007; Verma et al., 2009). While the role of glucose in the seminal fluid seems to be mainly related to spermatozoa membrane protection (Labbé and Maisse, 1996), the role of the other markers analyzed in the present study is still not clear. Although it is possible that triglycerides, and total cholesterol aid in SF viscosity, elevated concentrations can compromise sperm motility (Cosson, 2019). Therefore, the elevated concentrations observed in the present study (i.e., up to 210 mg dL-1 of triglycerides and up to 300 mg dL-1 of total cholesterol), may be related to other (possibly nutritional) functions, in addition to spermatozoa motility and protection in the face of environmental tonicity alterations. Either way, the presence of energy markers in the SF seems an evolutionary adaptation to ensure sperm quality, either by ensuring the integrity of sperm membranes or as an additional energy source for their mitochondria to optimize motility during fertilization.

We found moderate evidence for our second hypothesis that similar concentrations of all markers would be observed in the serum of both sexes. Although triglyceride concentrations were higher in the serum of pregnant females compared to sexually active males, the concentrations of the other markers were similar in both sexes. Such a pattern may indicate a greater importance of triglycerides for females during this period due to vitellogenesis that takes place prior to embryo development and requires great hepatic energy reserve mobilization. Similar concentrations of both cholesterol and ketone bodies in adults of both sexes have also been reported in Atlantic nurse sharks (*Ginglymostoma cirratum*) (Moorhead et al., 2021), and similar concentrations of total cholesterol in the serum of adult males and females were also reported for small-spotted catsharks (*Scyliorhinus canicula*) (Valls et al., 2016). In contrast, 3-β-hydroxybutyrate concentrations were higher in male *S. canicula*, attributed to higher physical activity compared to females. In both studies, differences in the analyzed markers were related to the period of the year, indicating that this variable imposes a greater influence (possibly related to the reproductive period) on these markers than sex itself.

We obtained mixed evidence for our third hypothesis that similar concentrations of all markers would be observed in both UL and SF. Although no statistically significant differences were noted between the two parental matrices for the analyzed energy markers, such a pattern may be a reflection of the low sample size of the present study. Furthermore, the lack of statistical differences between UL and SF may have been masked by the presence of outliers, which were not removed from the statistical analyses, as outliers in biological data may indicate the presence of distinct biological processes that define differential metabolic processes and are important to consider. Therefore, noted concentration trends will be briefly discussed. Overall, glucose concentrations were higher in the UL when compared to the SF, in a contrasting pattern to β-hydroxybutyrate. The same, however, was not observed for triglycerides and total cholesterol, as the concentrations of these markers were very similar in both parental matrices. High concentrations of some of the determined markers in UF and SF may indicate that each sex invest more with these markers, as some of them seem to be predominantly consumed in sperm fertilization/mobility and embryo nutrition. Furthermore, it is possible that energy demands for sperm fertilization/mobility may be similar to female investments in embryos, although no studies in this regard are available to date, requiring further assessments. For species for which this type of information is available, glucose concentrations in the UL were consistently low (Ornate Wobbegong shark, *Orectolobus ornatus*—10,8 mg dL^−1^; Spiny Dogfish, *Squalus acanthias*—0.001 mg dL^−1^; and Dusky Smoothhound *Mustelus canis*—0.0012 mg dL^−1^) (Lombardi et al., 1993; Ellis and Otway, 2011) or significantly higher (*Banded Houndshark, Triakis scyllium*—157 mg dL^−1^), contrasting with the intermediate values observed in the present study (72.58 ± 63.83 mg dL^−1^). Triglycerides in the UL have also been determined in *O. ornatus* (ranging from 0.03 to 0.93 mmol L^−1^) (Ellis and Otway, 2011), and cholesterol in *O. ornatus* (ranging from 9.6 to 27 mg dL^−1^) (Ellis and Otway, 2011), and in *T. scyllium* (47.3 ± 3.59 mg dL^−1^) (Minamikawa and Morisawa, 1996). Concentrations detected in the present study were remarkably higher for triglycerides (9.06 ± 4.13 mg dL^−1^), and slightly higher for cholesterol (34.41 ± 26.65 mg dL^−1^). The possible reasons why *P. horkelii* present higher values are a call for speculation, namely species-specific patterns, a more significant role of this energetic substrate in batoids when compared to sharks, or evidence that the UL presents an evolutionary adaptation for embryo nutrition in this species that does not occur in other elasmobranchs studied to date. Either way, our results highlight the complexity of UL in lecithotrophic viviparous species, emphasizing the need for additional studies to aid in our understanding of elasmobranch energy metabolism. To the best of our knowledge, data on the analyzed markers for elasmobranch SF are only available for *T. scyllium*, with mean glucose concentration of 12 mg dL^−1^, and mean cholesterol concentration of 38.3 mg dL^−1^ (Minamikawa and Morisawa, 1996). Both markers were detected in higher concentrations in *P. horkelii* SF (32.43 ± 39.07 mg dL^−1^ and 78.73 ± 133.93 mg dL^−1^, respectively), possibly for the same reasons cited above. Taken together, our results indicate that, while triglycerides and total cholesterol seem to present similar importance in both investigated parental matrices, glucose is found in higher concentrations in the UL and β-hydroxybutyrate in the SF, pointing to the greater relevance of each substrate in each of these parental fluids.

We also found mixed evidence for our fourth hypothesis, as statistical differences of circulating triglycerides and total cholesterol were observed between the UL and maternal serum. More specifically, both markers were lower in the UL. Similar results have been reported for *S. acanthias, M. cani*s, and the Atlantic Sharpnose Shark (*Rhizoprionodon terraenovae*), all presenting higher serum triglyceride levels when compared to the UL (Lombardi et al., 1993). Such a result may be indicative of a greater demand for mobilization of both markers, since in addition to the portion being directed to the developing litter, an extra portion of these substrates seems to be being mobilized for maternal use. This pattern is very interesting, as it indicates that both metabolites have a hitherto unknown relevance for *P. horkelii* females, not only for yolk production, but also for female daily activities during the gestational period. No other statistical differences were observed for the other evaluated makers between the UL and maternal serum, corroborating glucose and total cholesterol data from pregnant *T. scyllium* (Minamikawa and Morisawa, 1996), and in contrast with data available for *S. acanthias, M. cani*s, *R. terraenovae* and the Sandbar Shark (*Carcharhinus plumbeus*) (Lombardi et al., 1993), for which glucose concentrations were consistently higher in maternal serum compared to the UL. Such a difference may be attributed to distinct reproductive modes, as the aforementioned species (except for *T. scyllium*) are placental viviparous, possibly displaying a lower supply of metabolites *via* uterine fluid. Although no statistical differences were observed between SF and paternal serum, the circulating total cholesterol serum levels in males were slightly higher than that observed in SF, also corroborating data for sexually active male *T. scyllium* individuals (Minamikawa and Morisawa, 1996). It is possible that this difference is due to the role of total cholesterol in the production and transport of steroid hormones, possibly indicating, even if indirectly, high hormonal activity during the reproductive period.

Consistent with our fifth hypothesis, correlations between markers in both UL and SF were observed. For the UL of pregnant females, the strong positive correlation between glucose and triglycerides indicates that both markers exhibit energetic relevance for this parental matrix. High levels of glucose and triglycerides during pregnancy in elasmobranchs are, in fact, expected, as the energetic demands for embryo development must be balanced with the demands of other physiological processes vital to females (Wosnick, personal observation). In the case of such high energy demands, circulating β-hydroxybutyrate may not be sufficient, so glucose may be assuming a primary role along with triglycerides, as observed for other vertebrates (McWilliams, 2011). In addition, the positive correlation observed herein indicates that both compounds are being made available at similar rates to the offspring, at no direct cost to the embryos, as assimilation will not depend on gluconeogenesis. As for the SF of sexually active males, a strong positive correlation was detected between glucose and β-hydroxybutyrate, both final triglyceride hydrolysis products. Two additional negative correlations between glucose and triglycerides and between β-hydroxybutyrate and triglycerides were also detected, further indicating hydrolysis as the main physiological pathway for energy mobilization in this fluid. More specifically, with triglyceride hydrolysis, the circulating levels of this molecule will decrease, concomitantly with increases in its by-products (i.e., fatty acids and glycerol), allowing for the synthesis of ketone bodies and glucose. It is, however, important to emphasize that both UL and SF are merely fluids, with no described capacity to synthesize the markers evaluated in the present study. That being said, even if all markers are in fact being made available to sperm and embryos as energy sources, both matrices still depend on parental serum availability through the diet, and it is possible that the concentrations in these fluids are affected depending on feeding opportunities during the reproductive period. Interestingly, no correlation between markers was detected in the serum of either sex, or between the serum and each parental matrix. Although further studies are required to better understand these patterns, it is possible that the presence of marker correlations in the matrices and absence in the serum are related to greater stability of UL and SF compared to the more dynamic profile of serum, which reflects the short-term energetic state of an organism. Concerning the lack of correlations between the investigated markers in the serum and in the parental matrices in both sexes, further investigations are also needed, but it is possible to infer that, even if metabolite provision from the serum to matrices does occur, barriers in both female and male reproductive systems must be in place, ensuring greater stability and some degree of homeostasis for the parental matrices.

Lastly, we found partial support for our sixth hypothesis, as glucose appeared to be very significant to embryos, comprising one of the metabolite provisioning patterns detected in the UL of analyzed females. Interestingly, in a study performed with *T. scyllium*, sperm motility duration was longer in glucose-rich UF, suggesting the role of hexose in ensuring sperm motility in the female reproductive tract (Minamikawa and Morisawa, 1996). In the case of β-hydroxybutyrate, concentrations in both UL and SF were very similar to those observed in adult Atlantic nurse sharks during the mating period (Rangel et al., 2021), indicating that this molecule might be made available to the SF when serum levels are high. From an evolutionary point of view, making β-hydroxybutyrate and glucose available to embryos and spermatozoa would be advantageous, as it would make these compounds available without the need for lipid assimilation mechanisms that can be costly to the offspring. However, further studies are necessary to fully understand the role of both substrates prior to fertilization (sperm) and in the early stages of development (embryos), and if the patterns that are described for adults (β-hydroxybutyrate playing a primary role, and glucose not as important as for other vertebrates) are also observed in the offspring. Despite preliminary, our data reveal distinct patterns of marker concentrations in the investigated parental matrices that may provide insights into the use of energy sources by spermatozoa and developing embryos. Three patterns were noted in the UL, namely 1) the predominance of glucose, 2) the predominance of β-hydroxybutyrate and total cholesterol, and 3) reduced concentrations for all analyzed substrates. As all embryos were at similar stages of development (total length ranging from 210 to 240 mm), it is unlikely that the observed patterns are related to pregnancy progress, but rather are a result of maternal diet, which may directly influence serum concentrations, and consequently, UL composition. This is of particular relevance, as our data indicate that the UL seems to have a complementary role to the yolk and that any alteration in the maternal diet will alter the substrates made available to the offspring. In the case of SF, four distinct patterns were observed, namely 1) the predominance of triglycerides, 2) the predominance of total cholesterol, 3) the predominance of glucose, and β-hydroxybutyrate, and 4) intermediate concentrations for all energy substrates. In teleost fish, seminal fluid consists of organic and inorganic components. While the inorganic portion is responsible for sperm motility, the organic component is related to energy metabolism (Rurangwa et al., 2004), and its overall composition is influenced by several factors, including season (Aramli et al., 2013). In elasmobranchs, accessory Leydig glands synthesize proteins, resulting in elevated protein levels in the SF (Jones and Lin, 1993). Alkaline glands are also present near the seminal vesicle, being responsible for the production of a highly alkaline secretion linked to sperm production (Hamlett, 1999). Moreover, the SF of some sharks contains high concentrations of certain steroids (Simpson et al., 1964; Gottfried and Chieffi, 1967), which could partially explain the predominance of total cholesterol as a pattern. It is not clear if male serum composition affects SF composition as observed for the UL in pregnant females. However, it is plausible to infer that the serum may indeed play a role in seminal production, even if indirectly. Thus, it is also possible that the male diet affects sperm quality, not only in terms of motility through semen viscosity but also in terms of nutritional composition/availability to spermatozoa for oocyte fertilization.

Some important study limitations should account for in future research. First, as the studied species is listed as Critically Endangered, our sample size was low. Future studies should consider investigating these aspects in other elasmobranch species, aiming at more robust statistical analyses in easier to collect species. Second, also due to the difficult access to *P. horkelii* individuals, comparative analyses with juveniles, females, and adult males outside the reproductive period were not possible. Thus, future studies may also benefit from considering individuals at different life stages and adults outside the reproductive period. Third, our study evaluated the markers only in serum and reproductive matrices of pregnant females and sexually active males, and future studies may benefit from including other biological samples, such as the liver. Finally, considering the importance of the yolk for embryonic nutrition, future studies should include an analysis of markers in this matrix, in order to unravel additional patterns and correlations.

## Conclusion

The results presented herein indicate metabolite provisioning to sperm and developing embryos through parental matrices. The available concentrations seem to be influenced by female and male diets during the reproductive period, and triglycerides seem to have a greater importance in the energy metabolism of females during pregnancy when compared to sexually active males. While seminal fluid metabolites may present a protective function, it is possible that nutritional provision also occurs. Finally, evolutionary adaptations are also a possibility, as lecithotrophic viviparous species such as *P. horkelii* are expected to produce a very simple uterine liquid when compared to matrotrophic and placental species, although this was proven as erroneous, as organic molecules of great complexity were detected at very significant concentrations in the present study. Furthermore, passive exchange through the uterine wall without use by the offspring would represent an unnecessary energy expenditure, especially considering the complexity of reproductive modes observed in elasmobranchs.

## Data Availability

The raw data and statistical codes of the present study are available for consultation through the links: https://figshare.com/articles/dataset/Data/19701247, and https://figshare.com/articles/software/R_code/19700521.
